# Radiological Assessment of Sarcopenia and Its Association with Metabolic Markers in Patients with Liver Cirrhosis

**DOI:** 10.3390/jcm15134854

**Published:** 2026-06-23

**Authors:** Sedat Çiçek, Hasan Duyu, Selman Çetin, Abdulvahap Hohluoğlu, Furkan Kırsoy, Jehat Kılıç, Abdullah Mübin Özercan, Hakan Artaş, Mehmet Yalnız, İbrahim Halil Bahçecioğlu

**Affiliations:** 1 Department of Gastroenterology, Fırat University, 23119 Elazığ, Türkiye; 2 Department of Internal Medicine, Ergani State Hospital, 21950 Diyarbakır, Türkiye; 3 Department of Rheumatology, Fırat University, 23119 Elazığ, Türkiye; 4 Department of Radiology, Fırat University, 23119 Elazığ, Türkiye

**Keywords:** liver cirrhosis, sarcopenia, vitamin D deficiency, insulin-like growth factor 1

## Abstract

**Background**: Cirrhosis is a progressive liver disease often associated with sarcopenia. Vitamin D and IGF-1 alterations may contribute to muscle loss and disease progression. This study evaluated their relationship in cirrhotic patients. **Methods**: A total of 90 patients with liver cirrhosis were included in this retrospective observational study. Clinical and laboratory data were collected, and disease severity was assessed using Child–Pugh and MELD-Na scores. Sarcopenia was evaluated using CT-based skeletal muscle index at the L3 level with sex-specific cut-offs. Patients with malignancy, acute liver failure, recent surgery, or muscle-affecting conditions were excluded. Vitamin D and IGF-1 levels were classified using standard and age-adjusted reference ranges. **Results**: A total of 90 patients were included, of whom 42 were alive, and 48 died during follow-up. Gender distribution was similar between groups (*p* = 0.388). Skeletal muscle area was significantly lower in non-survivors (110 vs. 140 cm^2^, *p* = 0.002), while body mass index did not differ (*p* = 0.570). Vitamin D levels were significantly lower (10.0 vs. 17.9 ng/mL, *p* < 0.001), and hemoglobin levels were reduced in the non-survivor group (10.76 ± 2.13 vs. 12.87 ± 2.57 g/dL, *p* = 0.001). In multivariate analysis, age (OR 1.046, *p* = 0.032), MELD-Na score (OR 1.200, *p* = 0.001), and vitamin D level (OR 0.920, *p* = 0.024) were independently associated with mortality. **Conclusions**: CT-based sarcopenia assessment is a useful adjunct in cirrhosis when interpreted with disease severity. Radiological muscle depletion is common and associated with worse outcomes, while vitamin D deficiency independently associated with mortality, highlighting its potential as a biomarker and therapeutic target.

## 1. Introduction

Cirrhosis is a chronic liver disease characterized by progressive hepatocellular damage, widespread fibrosis, and regenerative nodule formation, and is associated with high morbidity and mortality. The progression and decompensation of cirrhosis are not limited to portal hypertension and hepatocellular insufficiency but result from complex, multifactorial pathophysiological processes in which systemic disorders such as sarcopenia also play a significant role [1]. Recent studies have demonstrated that the presence of sarcopenia in cirrhotic patients is associated with reduced quality of life and an increased risk of complications. Therefore, sarcopenia is considered a systemic indicator of hepatic reserve [2].

Sarcopenia is defined as a syndrome characterized by a reduction in skeletal muscle mass, muscle strength, and physical function. In patients with cirrhosis, sarcopenia has been reported to correlate with disease stage and severity [3]. Furthermore, the presence of sarcopenia has been shown to be associated with the development of decompensation, increased susceptibility to infections, q higher incidence of hepatic encephalopathy, increased hospitalization rates, and mortality [4,5]. The development of sarcopenia is a multifactorial process resulting from the interaction of various pathophysiological mechanisms, including protein–energy malnutrition, chronic inflammation with increased cytokine levels, hyperammonemia, hormonal imbalances (particularly disruptions in the testosterone and IGF-1 axis), physical inactivity, and portal hypertension [6]. Insulin-like growth factor-1 (IGF-1) is synthesized in the liver and exerts anabolic effects through the IGF-1/PI3K/Akt/mTOR pathways, which play a central role in muscle protein synthesis [7]. As liver cirrhosis progresses, the functional hepatocyte mass decreases, leading to reduced circulating IGF-1 levels. Consequently, muscle protein synthesis declines while muscle breakdown increases, potentially contributing to the development of sarcopenia. However, it remains unclear whether the relationship between IGF-1 levels and sarcopenia is independent of the severity of liver disease [8].

In addition, vitamin D is a hormone that exerts its effects through the vitamin D receptor in muscle cells and plays an important role in muscle function and quality [9]. Vitamin D deficiency is common in patients with cirrhosis and is associated with impaired hepatic 25-hydroxylation, cholestasis, malabsorption, and inadequate sunlight exposure. Experimental and clinical evidence suggests that vitamin D is involved in the regulation of myogenesis, muscle function, and inflammatory responses. Low serum 25-hydroxyvitamin D levels have been associated with the presence and severity of sarcopenia [10,11]. Although limited, some studies have demonstrated beneficial effects of vitamin D supplementation on muscle mass and strength. These findings suggest that vitamin D may serve as a potential prognostic biomarker and therapeutic target for sarcopenia in cirrhosis [12].

The present study aimed to assess radiologically defined sarcopenia in patients with liver cirrhosis and to investigate its association with metabolic markers, particularly vitamin D and IGF-1. Additionally, we evaluated the relationship between radiological muscle depletion and liver disease severity, cirrhosis-related complications, and clinical outcomes, including mortality.

## 2. Materials and Methods

A total of 90 patients who were followed for liver cirrhosis at our hospital between January 2020 and December 2024 were included in the study. The study was designed with a retrospective observational approach. All procedures were conducted in accordance with the principles of the Declaration of Helsinki. Ethical approval was obtained from the Institutional Ethics Committee of our hospital on 14.05.2026, with approval number 08-20.

Clinical and laboratory data were retrieved retrospectively from the hospital electronic medical record system, ENLIL Hospital Information Management System (ENLİL, Elazığ, Turkey). Demographic data, including age, sex, height, weight, and body mass index (BMI), were recorded for all patients. In addition, laboratory parameters such as white blood cell count (WBC), hemoglobin, platelet count, albumin, serum creatinine, total, direct, and indirect bilirubin levels, and the international normalized ratio (INR) were collected. Furthermore, disease severity was assessed using the Child–Pugh classification and the Model for End-Stage Liver Disease—Sodium (MELD-Na) score, which were documented for each patient.

### 2.1. Inclusion Criteria

- Age ≥ 18 years and <75 years (young-old included).
- Clinically confirmed liver cirrhosis.
- Availability of abdominal computed tomography (CT) imaging within ±3 months of the index date.
- Complete clinical and laboratory data.

### 2.2. Exclusion Criteria

- Active hepatocellular carcinoma or extrahepatic malignancy.
- Major surgical intervention within the past 6 months.
- Acute liver failure.
- Neuromuscular disease or systemic disease affecting muscle metabolism.
- CT images obtained during acute infection or during intensive care unit (ICU) stay.

**Figure 1:**
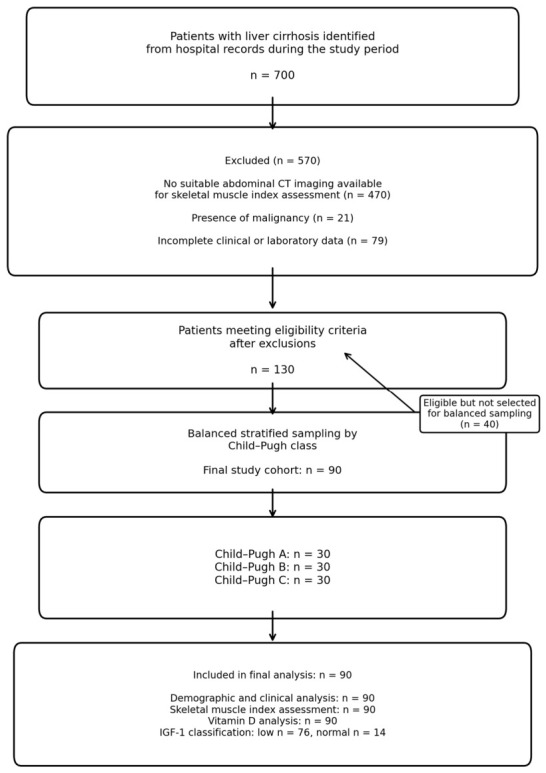
Flow Diagram of Patient Selection and Final Study Cohort.

For subgroup analyses, patients were categorized according to mortality status (survivors and non-survivors), sex (male and female), and liver disease severity based on the Child–Pugh classification (Child–Pugh A, B, and C). Clinical, laboratory, and radiological characteristics, including Skeletal Muscle Index (SMI) and metabolic markers, were compared between these groups to evaluate their associations with disease severity and clinical outcomes (Figure 1).

### 2.3. Study Population and Sampling

This retrospective observational study included patients with liver cirrhosis who underwent abdominal CT and laboratory evaluation between January 2020 and December 2024. After application of the predefined inclusion and exclusion criteria, 130 eligible patients were identified.

Because the primary objective of the study was to compare radiological sarcopenia and metabolic markers across different stages of liver disease severity, rather than to estimate their prevalence in the overall cirrhosis population, a balanced stratified sampling approach based on Child–Pugh classification was adopted. Child–Pugh A patients were substantially more numerous than Child–Pugh C patients among the eligible population. To reduce this imbalance and enable comparable analyses across disease severity groups, 30 patients from each Child–Pugh class (A, B, and C) were included in the final analytical cohort. Patient selection within each Child–Pugh stratum was performed using random sampling from the eligible population. Consequently, the final study population consisted of 90 patients.

### 2.4. Measurement of Skeletal Muscle Index

SMI is a quantitative parameter used to assess skeletal muscle mass and to identify sarcopenia in both clinical practice and research settings. Radiological muscle assessment is commonly derived from cross-sectional imaging techniques. In this study, computed tomography images were obtained using a Philips CT scanner (Philips Healthcare, Best, The Netherlands).

### 2.5. Method of Measurement

SMI is calculated using axial CT images obtained at the level of the third lumbar vertebra (L3). At this level, the cross-sectional area of skeletal muscle has been shown to strongly correlate with whole-body skeletal muscle mass.

Muscle groups included in the analysis typically comprise the psoas, erector spinae, quadratus lumborum, and the abdominal wall muscles (transversus abdominis, internal and external obliques, and rectus abdominis). Skeletal muscle area (SMA) is measured by manual or semi-automated segmentation using predefined Hounsfield unit (HU) thresholds, commonly ranging from −29 to +150 HU.

The SMI is calculated by normalizing the total SMA to the square of the patient’s height, using the following formula:

SMI (cm^2^/m^2^) = SMA at L3 (cm^2^)/height^2^ (m^2^).

### 2.6. Clinical Interpretation

Although extremity muscles may be influenced by physical activity, the psoas and abdominal muscle groups are relatively independent of physical activity levels. Therefore, muscle loss can be reliably assessed on CT images obtained at the third and fourth lumbar vertebral (L3–L4) levels. In CT-based measurements, SMI values below 38.5 cm^2^/m^2^ in women and 52.4 cm^2^/m^2^ in men are considered diagnostic for radiological muscle depletion [13,14,15]. The CT-based cut-off values used to define low skeletal muscle mass were adopted from the study by Montano-Loza et al., as these thresholds have been widely applied in studies evaluating body composition in patients with liver cirrhosis [15].

### 2.7. Evaluation of Vitamin D and IGF-1

Vitamin D levels were categorized into four clinically relevant groups as follows:

<10 ng/mL: severe deficiency.
10–20 ng/mL: deficiency.
20–30 ng/mL: insufficiency.
>30 ng/mL: normal vitamin D status.

In addition, vitamin D deficiency was analyzed as a binary variable, with values <30 ng/mL classified as deficient and values ≥ 30 ng/mL classified as non-deficient. Serum IGF-1 levels were categorized as low or normal according to age-adjusted reference ranges provided by the performing laboratory. Because circulating IGF-1 concentrations decline with age, the lower limit of normal was defined as approximately 116 ng/mL for individuals aged 20–29 years, 109 ng/mL for those aged 30–39 years, 95 ng/mL for those aged 40–49 years, 71 ng/mL for those aged 50–59 years, 52 ng/mL for those aged 60–69 years, and 34 ng/mL for individuals aged 70 years and older. Values below the corresponding age-specific lower reference limit were classified as low IGF-1 levels.

### 2.8. Statistical Analysis

Continuous variables were assessed for normality using visual methods (histograms and Q–Q plots) and analytical tests. Normally distributed variables were expressed as mean ± standard deviation, whereas non-normally distributed variables were presented as median (minimum–maximum). Comparisons among Child–Pugh classes were performed using one-way analysis of variance (ANOVA) for normally distributed variables and the Kruskal–Wallis test for non-normally distributed variables. When significant differences were identified, post hoc pairwise comparisons were conducted using Tukey’s test or Games–Howell test, as appropriate. Categorical variables were compared using the chi-square test or Fisher’s exact test.

Univariate logistic regression analysis was performed to identify variables associated with mortality. Variables with clinical relevance or statistical significance in univariate analyses were entered into a multivariable logistic regression model. Odds ratios (ORs) and 95% confidence intervals (CIs) were reported. Multicollinearity was assessed using tolerance and variance inflation factor (VIF) values. The calibration of the multivariable logistic regression model was assessed using the Hosmer–Lemeshow goodness-of-fit test. Receiver operating characteristic (ROC) curve analysis was performed to evaluate the predictive performance of serum vitamin D levels for mortality, and the area under the curve (AUC) was calculated. A two-sided *p*-value <0.05 was considered statistically significant. Statistical analyses were performed using IBM SPSS Statistics version 26.0 (IBM Corp., Armonk, NY, USA).

## 3. Results

A total of 90 patients with liver cirrhosis were included in this study. The mean age was 59.2 ± 13.4 years, and the majority of patients were male (*n* = 55, 61.1%). Chronic hepatitis B was the most common etiology of cirrhosis (*n* = 45, 50.0%), followed by cryptogenic cirrhosis (*n* = 19, 21.1%) and hepatitis B with delta coinfection (*n* = 9, 10.0%). Other etiologies included primary biliary cholangitis (*n* = 5, 5.6%), autoimmune hepatitis (*n* = 4, 4.4%), alcohol-related liver disease (*n* = 2, 2.2%), and miscellaneous causes (*n* = 6, 6.7%). The mean MELD-Na score was 14.6 ± 7.4. According to the Child–Pugh classification, 30 patients (33.3%) were classified as Child–Pugh A, 30 (33.3%) as Child–Pugh B, and 30 (33.3%) as Child–Pugh C. Decompensated cirrhosis was present in 58 patients (64.4%), while 32 patients (35.6%) had compensated disease. Ascites was absent in 35 patients (38.9%), mild in 26 (28.9%), and severe in 29 (32.2%).

The mean SMI was 43.5 ± 7.6 cm^2^/m^2^, and radiological muscle depletion was identified in 59 patients (65.6%). The mean serum vitamin D level was 14.5 ± 8.3 ng/mL. Only four patients (4.4%) had vitamin D levels above 30 ng/mL, whereas 16 (17.8%) had levels between 20 and 30 ng/mL, 38 (42.2%) had levels between 10 and 20 ng/mL, and 32 (35.6%) had levels below 10 ng/mL. IGF-1 levels were low in 76 patients (84.4%) and normal in 14 patients (15.6%). The mean serum creatinine level was 1.01 ± 0.75 mg/dL, the mean total bilirubin level was 3.19 ± 5.63 mg/dL, and the mean INR was 1.44 ± 0.61. During follow-up, mortality occurred in 43 patients (47.8%), while 47 patients (52.2%) survived (Table 1).

Baseline clinical, laboratory, and body composition characteristics according to mortality status are summarized in Table 2. A total of 90 patients were included, of whom 42 survived and 48 died during follow-up. Gender distribution did not differ significantly between survivors and non-survivors (male/female: 28/14 vs. 27/21, *p* = 0.388). Non-survivors were significantly older than survivors (median age 66.5 vs. 54 years, *p* < 0.001). Liver disease severity was significantly associated with mortality. Non-survivors had higher MELD-Na scores than survivors (18 [8–39] vs. 9.5 [6–28], *p* < 0.001). In addition, Child–Pugh class distribution differed significantly between groups (*p* < 0.001), with most survivors classified as Child–Pugh A (59.5%) and most non-survivors as Child–Pugh C (54.2%).

Markers of body composition differed notably between groups. SMA was significantly lower in non-survivors than in survivors (median 110 cm^2^ vs. 140 cm^2^, *p* = 0.002), and SMI was also lower in non-survivors (41.37 ± 6.96 vs. 45.96 ± 7.69 cm^2^/m^2^, *p* = 0.004). In contrast, body mass index did not differ significantly between groups (*p* = 0.570).

Among laboratory parameters, serum vitamin D levels were significantly lower in non-survivors (median 10.0 vs. 17.9 ng/mL, *p* < 0.001), whereas vitamin B12 levels were significantly higher (median 548 vs. 423 pg/mL, *p* = 0.011). Hemoglobin levels were significantly lower in non-survivors than in survivors (10.76 ± 2.13 vs. 12.87 ± 2.57 g/dL, *p* = 0.001). No significant differences were observed in IGF-1, creatinine, platelet count, white blood cell count, or AFP levels between groups (all *p* > 0.05), although AFP showed greater variability among non-survivors. Overall, lower SMI, lower serum vitamin D levels, anemia, and older age were associated with mortality, whereas BMI and most routine laboratory parameters did not differ significantly between survivors and non-survivors (Table 2).

A statistically significant association was found between gender and radiological muscle depletion status. Radiological muscle depletion was more prevalent among males compared to females (76.4% vs. 57.1%), whereas the absence of radiological muscle depletion was more common in females (42.9% vs. 23.6%) (*p* < 0.001). No significant association was observed between gender and vitamin D status. The majority of participants in both groups had insufficient vitamin D levels (94.5% in males vs. 97.1% in females), with comparable proportions of deficiency (5.5% vs. 2.9%, respectively; *p* = 0.560). Similarly, mortality rates did not differ significantly by gender. Although the proportion of non-survivor individuals was higher among females than males (60.0% vs. 49.1%), this difference was not statistically significant (*p* = 0.312) (Table 3).

Baseline characteristics differed significantly across Child–Pugh classes. Patients with Child B and C were older than those with Child A, with a significant difference between Child A and B (*p* = 0.001). Hemoglobin levels progressively decreased with worsening Child class and were significantly lower in Child C compared with Child A (*p* < 0.001). White blood cell counts were higher in Child C than in Child A and B (both *p* = 0.010). No significant differences were observed in BMI, platelet count, or creatinine levels among groups.

Markers of liver dysfunction, including albumin, total bilirubin, INR, and MELD-Na score, showed significant deterioration with increasing Child–Pugh class (all *p* < 0.001). SMA and SMI were significantly lower in Child–Pugh B and C patients than in Child–Pugh A patients (*p* < 0.001), whereas no significant differences were observed between Child–Pugh B and C (Table 4).

In univariate analysis, age, hemoglobin level, MELD-Na score, SMI, SMA, and vitamin D level were significantly associated with mortality (all *p* < 0.01). In contrast, gender, platelet count, IGF-1, and creatinine were not significantly associated with mortality. In multivariate logistic regression analysis, age (OR 1.046, 95% CI 1.004–1.091; *p* = 0.032), MELD-Na score (OR 1.200, 95% CI 1.080–1.335; *p* = 0.001), and vitamin D level (OR 0.920, 95% CI 0.856–0.989; *p* = 0.024) remained independently associated with mortality. Hemoglobin showed a borderline association after adjustment (*p* = 0.097), while SMI was not independently associated with mortality (Table 5). Collinearity diagnostics for the initial and final mortality regression models and calibration assessment using the Hosmer–Lemeshow goodness-of-fit test are provided in the Supplementary Materials (Tables S1–S3).

**Figure 2:**
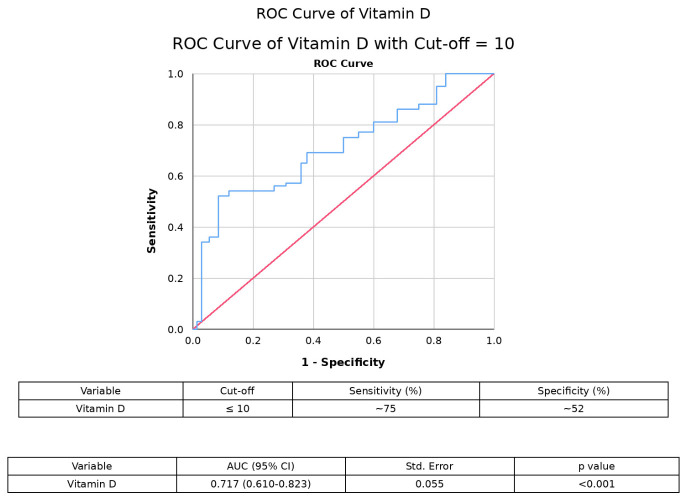
Receiver operating characteristic (ROC) curve of vitamin D for mortality prediction.

ROC curve analysis demonstrated that serum vitamin D had a fair discriminatory ability for mortality, with an area under the curve (AUC) of 0.717 (95% CI: 0.610–0.823; *p* < 0.001). Using ROC curve coordinates, a cut-off value of ≤10 was identified as clinically relevant. At this threshold, vitamin D showed a sensitivity of 75% and a specificity of 52%, indicating moderate sensitivity with acceptable specificity (Figure 2).

## 4. Discussion

In this study, we aimed to evaluate radiological muscle depletion, vitamin D levels, IGF-1 levels, and other clinical and biochemical parameters in patients with cirrhosis. We also investigated the relationships of these parameters with disease severity, clinical course, and mortality. We also sought to investigate the association between radiological muscle depletion, disease stage, and clinical course and to clarify its potential impact on prognosis. Our findings are consistent with the existing literature, demonstrating that radiological muscle depletion is common in cirrhotic patients [16]. Although the SMI was significantly lower in patients who died and was associated with mortality in univariate analysis, this association was not maintained after multivariable adjustment. A possible explanation is that the prognostic effect of reduced muscle mass is partially mediated through overall liver disease severity. Patients with advanced cirrhosis frequently exhibit both higher MELD-Na scores and more pronounced muscle wasting, resulting in overlapping prognostic information between these variables. Consequently, after adjustment for age and MELD-Na, the independent contribution of SMI to mortality was attenuated. Nevertheless, the significantly lower SMA and SMI observed among non-survivors suggest that radiological muscle depletion remains closely linked to adverse clinical outcomes and may serve as a marker of advanced disease burden.

The lack of a significant difference in body mass index between groups may be explained by fluid retention commonly observed in cirrhosis, such as ascites and edema, which can distort body weight measurements. This limitation reduces the reliability of BMI in reflecting true muscle mass and may lead to under-recognition of sarcopenia. Therefore, these findings support the notion that anthropometric measures alone may be insufficient, whereas cross-sectional imaging-based assessments, such as the SMA and SMI, provide a more sensitive evaluation of sarcopenia [17].

The relationship between disease severity and muscle mass was clearly demonstrated in our study. The significant decrease in the SMA and SMI with increasing Child–Pugh scores suggests that radiological muscle depletion is closely associated with the progression of cirrhosis. This finding supports the notion that sarcopenia cannot be explained solely by malnutrition, but rather reflects complex pathophysiological processes involving chronic inflammation, hormonal dysfunction, and metabolic disturbances [18]. Recent evidence suggests that hyperammonemia plays a central role in the development of sarcopenia in cirrhosis. Elevated ammonia levels increase skeletal muscle myostatin expression through NF-κB activation, thereby suppressing protein synthesis and promoting muscle proteolysis. In addition, hyperammonemia impairs mitochondrial function, leading to reduced ATP production, increased oxidative stress, and accelerated muscle catabolism. These findings indicate that myostatin upregulation and mitochondrial dysfunction are key mechanisms linking hyperammonemia to muscle loss in cirrhotic patients [19,20].

Our findings regarding the relationship between gender and radiological muscle depletion, mortality, and vitamin D levels are consistent with the existing literature. The higher prevalence of radiological muscle depletion in males has been attributed to hormonal differences and lifestyle-related factors. In addition, the lack of a significant difference in vitamin D levels between genders is in line with studies reporting similar vitamin D status in comparable populations. Although mortality rates were higher in females, this difference did not reach statistical significance, which may be explained by the multifactorial nature of mortality [21].

When evaluated in terms of metabolic markers, it is noteworthy that vitamin D levels were not only associated with mortality but also remained independently associated in multivariate analysis. Furthermore, the findings from ROC analysis suggest that vitamin D may serve as a potential biomarker for clinical risk stratification. Vitamin D remained independently associated with mortality after multivariable adjustment; however, this finding should not be interpreted as evidence of a causal relationship. Instead, vitamin D deficiency may reflect the combined effects of malnutrition, impaired liver function, chronic inflammation, reduced sunlight exposure, and other factors associated with advanced cirrhosis. Consequently, vitamin D deficiency may represent a marker of advanced disease burden and poor overall clinical status in patients with cirrhosis. Previous studies have similarly reported associations between low vitamin D levels and adverse clinical outcomes, including increased risk of infections and higher mortality in this population [22]. Therefore, monitoring vitamin D levels in clinical practice and providing supplementation, when necessary, may contribute significantly to patient management. In contrast, the lack of a significant association between IGF-1 levels and mortality suggests that the prognostic value of this parameter alone may be limited. This finding indicates that IGF-1 levels in cirrhosis are likely regulated by complex and multifactorial mechanisms.

In our study, the independent association of age and MELD-Na score with mortality supports their consistency with established prognostic models. The MELD-Na score is a well-validated tool widely used to predict mortality risk in patients with advanced liver disease, reflecting both hepatic dysfunction and systemic complications. Similarly, age has been consistently identified as an important determinant of clinical outcomes, likely due to reduced physiological reserve and increased comorbidity burden. These findings reinforce the robustness of current prognostic frameworks and highlight the importance of integrating both clinical and laboratory parameters in risk stratification [23].

Taken together, these findings suggest that radiological muscle depletion in cirrhosis represents not merely a complication but a systemic condition that may be associated with disease progression and survival. In particular, the independent prognostic value of vitamin D deficiency indicates that it may serve as a clinically modifiable target. However, prospective randomized controlled studies are needed to evaluate the clinical impact of vitamin D supplementation [24].

This study has several limitations. First, its retrospective single-center design may have introduced selection bias and limited the generalizability of the findings. Second, although mortality was evaluated as a clinical outcome, detailed time-to-event data, follow-up duration, and censoring information were not consistently available because of the retrospective nature of the study; therefore, survival analyses such as Cox proportional hazards regression could not be performed. Third, sarcopenia was assessed using CT-derived SMI without accompanying measures of muscle strength or physical performance, which are recommended by contemporary consensus definitions. Consequently, our findings should be interpreted in the context of radiologically assessed low muscle mass rather than fully characterized sarcopenia. Fourth, the CT-derived SMI cut-off values used in this study were adopted from the widely cited work of Montano-Loza et al. [15]. Although these thresholds have been extensively used in cirrhosis research, they were originally derived from oncology populations, and universally accepted cirrhosis-specific CT cut-offs are still lacking. Fifth, vitamin D levels were measured at a single time point, and information regarding seasonal variation, supplementation status, and longitudinal changes in vitamin D concentrations was not available. Sixth, the relatively small sample size and limited number of mortality events may have increased the risk of model overfitting despite efforts to minimize multicollinearity and restrict the number of variables included in multivariable analyses. Finally, the predictive models were not externally validated, and therefore the findings should be confirmed in larger prospective multicenter studies.

## 5. Conclusions

CT-based assessment of sarcopenia is a useful adjunct in cirrhosis but should be interpreted alongside disease severity. Radiological muscle depletion was highly prevalent, increased with disease progression, and was associated with mortality in unadjusted analyses but did not remain independently associated after multivariable adjustment. Lower vitamin D levels were associated with adverse outcomes in this cohort; however, its clinical utility as an independent prognostic biomarker remains uncertain and requires confirmation in prospective studies with comprehensive adjustment for potential confounders. However, the observational nature of this study precludes conclusions regarding causality or therapeutic benefit.

## Figures and Tables

**Table 1:** Baseline Characteristics of Study Population (*n* = 90).

Variable	Value
Demographic Characteristics	
Age, years	59.2 ± 13.4
Male sex	55 (61.1)
Female sex	35 (38.9)
Etiology of Cirrhosis	
Chronic Hepatitis B	45 (50.0)
Hepatitis B + Delta	9 (10.0)
Autoimmune Hepatitis	4 (4.4)
Primary Biliary Cholangitis	5 (5.6)
Alcohol-Related Liver Disease	2 (2.2)
Cryptogenic Cirrhosis	19 (21.1)
Other Causes *	6 (6.7)
Disease Severity	
MELD-Na score	14.6 ± 7.4
Child–Pugh A	30 (33.3)
Child–Pugh B	30 (33.3)
Child–Pugh C	30 (33.3)
Compensated Cirrhosis	32 (35.6)
Decompensated Cirrhosis	58 (64.4)
No Ascites	35 (38.9)
Mild Ascites	26 (28.9)
Severe Ascites	29 (32.2)
Body Composition	
SMI (cm^2^/m^2^)	43.5 ± 7.6
Radiological muscle depletion Present	59 (65.6)
Radiological muscle depletion Absent	31 (34.4)
Laboratory Parameters	
Vitamin D (ng/mL)	14.5 ± 8.3
>30 ng/mL	4 (4.4)
20–30 ng/mL	16 (17.8)
10–20 ng/mL	38 (42.2)
<10 ng/mL	32 (35.6)
IGF-1 levels (Normal-Low)	
Normal	14 (15.6)
Low	76 (84.4)
Creatinine (mg/dL)	1.01 ± 0.75
Total Bilirubin (mg/dL)	3.19 ± 5.63
INR	1.44 ± 0.61
Outcomes	
Survived	42 (46.7)
Died	48 (53.3)

Abbreviations: MELD-Na, Model for End-Stage Liver Disease-Sodium; SMI, Skeletal muscle index; INR, International Normalized Ratio; IGF-1, Insulin-like Growth Factor-1; PBC, Primary Biliary Cholangitis. * Other causes included polycystic liver disease, cardiogenic liver disease, Wilson disease, and metabolic dysfunction-associated steatotic liver disease (MASLD).

**Table 2:** Baseline clinical, laboratory, and body composition characteristics according to mortality status.

Variable	Survivors (*n* = 42)	Non-Survivors (*n* = 48)	*p* Value
Gender (Male/Female)	28/14	27/21	0.388
Age (Years)	54 ±12.5	66.5 (26–80)	0.001
Child–Pugh class, *n* (%)			<0.001
Child–Pugh A	25 (59.5)	5 (10.4)	
Child–Pugh B	13 (31.0)	17 (35.4)	
Child–Pugh C	4 (9.5)	26 (54.2)	
MELD–Na score	9.5 (6–28)	18 (8–39)	<0.001
SMA (cm^2^)	140 (88–192)	110 (76–157)	0.002
SMI (cm^2^/m^2^)	45.96 ± 7.69	41.37 ± 6.96	0.004
Vitamin D (ng/mL)	17.9 (5.6–36.5)	10.0 (2.3–45.0)	<0.001
IGF-1 (ng/mL)	97.6 ± 44.0	66.6 ± 32.9	0.129
Body mass index (kg/m^2^)	24.46 ± 3.15	24.86 ± 3.51	0.570
Creatinine (mg/dL)	0.80 (0.28–1.56)	0.83 (0.36–4.95)	0.549
Vitamin B12 (pg/mL)	423 (157–1461)	548 (253–2000)	0.011
Platelet count (×10^9^/L)	92.5 (22–439)	88 (23–326)	0.805
Hemoglobin (g/dL)	12.87 ± 2.57	10.76 ± 2.13	0.001
White blood cell count (×10^9^/L)	4.24 (1.89–16.62)	5.35 (1.50–18.30)	0.183
AFP (ng/mL)	3.25 (0.10–63.7)	2.40 (0.00–79,033)	0.072

Abbreviations: AFP, alpha-fetoprotein; SMA, Skeletal Muscle Area; SMI, Skeletal muscle index; BMI, body mass index; IGF-1, insulin-like growth factor-1; PLT, platelet count; WBC, white blood cell count; MELD-Na, Model for End-Stage Liver Disease–Sodium score.

**Table 3:** Association of Gender with Radiological Muscle Depletion, Mortality, and Vitamin D Levels.

Variable	Category	Male (*n* = 55)	Female (*n* = 35)	Total (*n*)	*p*-Value
Radiological Muscle Depletion	Absent	13 (23.6%)	15 (42.9%)	28	<0.001
	Present	42 (76.4%)	20 (57.1%)	62	
Vitamin D deficiency	Present	52 (94.5%)	34 (97.1%)	86	0.560
	Absent	3 (5.5%)	1 (2.9%)	4	
Mortality	Alive	28 (50.9%)	14 (40.0%)	42	0.312
	Deceased	27 (49.1%)	21 (60.0%)	48	

**Table 4:** Baseline Clinical, Laboratory, and Muscle Characteristics According to Child–Pugh Class.

Variable	Child A	Child B	Child C	*p* Value	Post Hoc Comparisons (Adjusted *p*)
Age (years)	56 (25–75)	64.5 (26–75)	66 (45–75)	0.007	A–B: 0.001; A–C: 0.195; B–C: 0.054 *
BMI (kg/m^2^)	24.9 ± 3.5	24.0 ± 3.1	25.1 ± 3.2	0.393	A–B: 0.934; A–C: 1.000; B–C: 0.575 *
WBC (×10^3^/µL)	4.3 (1.9–11.7)	4.1 (1.5–16.6)	5.7 (2.0–18.3)	0.022	A–B: 0.054; A–C: 0.010; B–C: 0.010
Hemoglobin (g/dL)	12.9 ± 2.7	11.4 ± 2.2	10.4 ± 2.2	0.001	A–B: 0.054; A–C: <0.001; B–C: 0.312 *
Platelet (×10^3^/µL)	93 (22–326)	74 (36–439)	105 (23–306)	0.340	—
Creatinine (mg/dL)	0.78 (0.28–2.20)	0.78 (0.36–1.48)	1.11 (0.38–4.95)	0.086	—
Albumin (g/dL)	4.0 ± 0.5	3.1 ± 0.5	2.5 ± 0.3	0.001	—
Total bilirubin (mg/dL)	0.95 (0.3–2.9)	1.35 (0.4–6.5)	3.75 (0.3–33.2)	0.001	—
INR	1.16 ± 0.16	1.24 (1.02–2.57)	1.60 (1.10–4.23)	0.001	—
MELD-Na	9 (6–22)	11 (8–21)	22 (9–39)	0.001	—
SMA (cm^2^)	139.3 ± 29.1	101.5 (76–182)	118.3 ± 25.4	0.001	A–B: 0.001; A–C: 0.009; B–C: 0.122
SMI (cm^2^/m^2^)	47.4 ± 6.6	40.3 ± 6.6	42.2 ± 7.4	0.001 *	A–B: 0.001; A–C: 0.011; B–C: 0.886

Abbreviations: BMI, body mass index; WBC, white blood cell count; SMA, Skeletal muscle area; SMI, Skeletal muscle index; INR, international normalized ratio; MELD-Na, Model for End-Stage Liver Disease–Sodium score. *: Kruskal–Wallis test.

**Table 5:** Univariate and multivariate logistic regression analyses of factors associated with mortality.

Variable	Univariate OR (95% CI)	*p* Value	Multivariate OR (95% CI)	*p* Value
Age (years)	1.064 (1.025–1.104)	0.001	1.046 (1.004–1.091)	0.032
Gender (male)	0.643 (0.272–1.517)	0.313	—	—
Hemoglobin (g/dL)	0.692 (0.568–0.843)	<0.001	0.797 (0.610–1.042)	0.097
Platelet count (×10^9^/L)	1.000 (1.000–1.000)	0.817	—	—
MELD-Na score	1.229 (1.112–1.358)	0.001	1.200 (1.080–1.335)	0.001
Vitamin B12 (pg/mL)	1.002 (1.000–1.003)	0.041	—	—
Creatinine (mg/dL)	2.520 (0.997–6.370)	0.051	—	—
SMI (cm^2^/m^2^)	0.917 (0.863–0.976)	0.006	0.980 (0.898–1.070)	0.657
SMA (cm^2^)	0.973 (0.957–0.989)	0.001	—	—
IGF-1 (ng/mL)	0.977 (0.947–1.008)	0.142	—	—
Vitamin D (ng/mL)	0.907 (0.852–0.965)	0.002	0.920 (0.856–0.989)	0.024

Abbreviations: CI, confidence interval; HGB, hemoglobin; IGF-1, insulin-like growth factor-1; OR, odds ratio; PLT, platelet count; SMA, Skeletal Muscle Area; SMI, skeletal muscle index. The multivariable logistic regression model demonstrated acceptable calibration according to the Hosmer–Lemeshow goodness-of-fit test (χ^2^ = 9.098, df = 8, *p* = 0.334).

## Data Availability

The data presented in this study are available on reasonable request from the corresponding author. The data are not publicly available due to privacy and ethical restrictions.

## Supplementary Materials

The following supporting information can be downloaded at: https://www.mdpi.com/article/10.3390/jcm15134854/s1, Table S1: Collinearity Diagnostics of Variables Included in the Mortality Regression Model, Table S2: Collinearity Diagnostics of the Final Mortality Regression Model, Table S3: Calibration of the multivariable logistic regression model assessed by the Hosmer–Lemeshow goodness-of-fit test.

## Abbreviations

The following abbreviations are used in this manuscript: AFP—Alpha-fetoprotein, ANOVA—Analysis of Variance, BMI—Body Mass Index, CI—Confidence Interval, CT—Computed Tomography, HU—Hounsfield Unit, IGF-1—Insulin-like Growth Factor-1, INR—International Normalized Ratio, MELD-Na—Model for End-Stage Liver Disease–Sodium, OR—Odds Ratio, ROC—Receiver Operating Characteristic, SD—Standard Deviation, SMI—Skeletal Muscle Index, SMA—Skeletal Muscle Area, and WBC—White Blood Cell Count.
