# Supplementary material for: Radiological Assessment of Sarcopenia and Its Association with Metabolic Markers in Patients with Liver Cirrhosis
Source: J Clin Med. 2026 Jun 23;15(13):4854. doi: 10.3390/jcm15134854 (PMC13361375; doi:10.3390/jcm15134854)
Supplement: Supplementary file 1 [file jcm-15-04854-s001.zip › jcm-4360245-supplementary.pdf]

Table S1. Collinearity Diagnostics of Variables Included in the Mortality Regression Model.

Variable	Tolerance	VIF	Interpretation
**Age (years)**	0.507	1.972	Acceptable
**HGB (gr/dL)**	0.375	2.667	Acceptable
**PLT (×10⁹/L)**	0.314	3.188	Acceptable / mild correlation
**MELD-Na**	0.513	1.948	Acceptable
**Vitamin B12 (pg/mL)**	0.325	3.078	Acceptable / mild correlation
**Creatinine (mg/dL)**	0.386	2.590	Acceptable
**SMI (cm²/m²)**	0.037	27.394	Severe multicollinearity
**SMA (cm²)**	0.043	23.030	Severe multicollinearity
**IGF-1 (ng/mL)**	0.694	1.441	Acceptable
**Vitamin D (ng/mL)**	0.366	2.732	Acceptable

Abbreviations: VIF, variance inflation factor; SMI, Skeletal muscle index; SMA, Skeletal Muscle Area; PLT, platelet count; B12, vitamin B12; IGF-1, insulin-like growth factor-1. Tolerance values <0.10 and VIF values >10 were considered indicative of significant multicollinearity.After exclusion of skeletal muscle surface area due to collinearity with SMI, the final model demonstrated acceptable collinearity statistics, with VIF values ranging from 1.391 to 2.953.

Table S2. Collinearity Diagnostics of the Final Mortality Regression Model

Variable	Tolerance	VIF	Interpretation
**Age (years)**	0.510	1.959	Acceptable
**HGB (gr/dL)**	0.385	2.599	Acceptable
**PLT (×10⁹/L)**	0.525	1.905	Acceptable
**MELD-Na**	0.522	1.917	Acceptable
**Vitamin B12 (pg/mL)**	0.357	2.802	Acceptable
**Creatinine (mg/dL)**	0.652	1.535	Acceptable
**SMI (cm²/m²)**	0.339	2.953	Acceptable
**IGF-1 (ng/mL)**	0.719	1.391	Acceptable
**Vitamin D (ng/mL)**	0.606	1.649	Acceptable

Abbreviations: VIF, variance inflation factor; PLT, platelet count; B12, vitamin B12; IGF-1, insulin-like growth factor-1; SMI, skeletal muscle index; MELD-Na, Model for End-Stage Liver Disease–Sodium score. Collinearity diagnostics were performed before multivariable regression analysis. In the final model, all tolerance values were above 0.10 and all VIF values were below 5, indicating no significant multicollinearity among the included variables. Therefore, age, hemoglobin, platelet count, MELD-Na, vitamin B12, creatinine, skeletal muscle index, IGF-1, and vitamin D were considered suitable for inclusion in the final mortality regression model.

Table S3. Calibration of the multivariable logistic regression model assessed by the Hosmer–Lemeshow goodness-of-fit test.

Statistic	Value
Chi-square (χ^2)	9.098
Degrees of freedom (df)	8
P-value	0.334

Interpretation: The Hosmer–Lemeshow goodness-of-fit test demonstrated acceptable calibration of the multivariable logistic regression model ($p > 0.05$), indicating no evidence of significant disagreement between observed and predicted mortality probabilities.
